# Identification of polymorphic loci in the deiodinase 2 gene and their associations with head dimensions in geese

**DOI:** 10.5713/ab.21.0382

**Published:** 2021-10-29

**Authors:** Yan Deng, Qian Hu, Bincheng Tang, Qingyuan Ouyang, Shenqiang Hu, Bo Hu, Jiwei Hu, Hua He, Guohong Chen, Jiwen Wang

**Affiliations:** 1 Farm Animal Genetic Resources Exploration and Innovation Key Laboratory of Sichuan Province, Sichuan Agricultural University, Chengdu 611130, Sichuan, China; 2 Key Laboratory of Animal Genetics and Breeding and Molecular Design of Jiangsu Province, Yangzhou University, Yangzhou 225000, Jiangsu, China

**Keywords:** Deiodinase 2, Geese, Head Dimensions, Knob, Polymorphic Loci

## Abstract

**Objective:**

This study was conducted to clone and compare the molecular characteristics of the deiodinase 2 (*DIO2*) gene between Sichuan White geese and Landes geese, and to analyze the association between polymorphisms of the *DIO2* gene and head dimensions in Tianfu meat geese.

**Methods:**

The coding sequence of the *DIO2* gene was cloned by polymerase chain reaction and vector ligation and aligned by DNAMAN software. A total of 350 Tianfu meat geese were used to genotype the polymorphisms of the *DIO2* gene and measure the head dimensions. Association analysis between the polymorphisms of the *DIO2* gene and head dimensions was carried out.

**Results:**

An 840-bp coding sequence of the *DIO2* gene was obtained and comparison analysis identified four polymorphic loci between Sichuan White geese and Landes geese. Further analysis showed that the dominant alleles for the four polymorphic loci were G, G, A, and T and the frequency of the heterozygous genotype was higher than that of the homozygous genotype in Tianfu meat geese. Compared to that in the population of non-knob geese of Tianfu meat geese, the head dimensions in the population of knob geese were significantly higher except for nostril height. However, in the non-knob geese, beak width 1, beak width 2, nostril length, cranial width 1, and maxillary length had significant differences among different genotypes or haplotypes/diplotypes.

**Conclusion:**

These results suggested that polymorphisms of the *DIO2* gene could be considered molecular markers to select larger heads of geese in the population of non-knob geese.

## INTRODUCTION

Deiodinase 2 (*DIO2*), predominantly converts prohormone T4 to active hormone T3, which plays important roles in several biological processes, such as the development of skeletal muscle, regulation of the hypothalamus-pituitary-thyroid axis, adaptive thermogenesis and metabolic control, behavior, and mood [1]. Evidence has shown that knockdown of the *DIO2* gene could result in increased fat storage in adipose tissue, hepatic steatosis [2], and increased subchondral bone mineral content [3]. Bone derived from DIO2-knockout mice showed reduced toughness, was brittle and had increased vulnerability to fracture, emphasizing that *DIO2* was an important prerequisite for optimal bone remodeling [4]. Notably, common variation in the *DIO2* gene, particularly the Thr92Ala substitution, was associated with osteoarthritis and intelligence quotient [5–7]. The subjects carrying the DIO2-Thr92Ala polymorphism had consistently lower femoral neck and total hip densities than wild-type subjects, accompanied by significantly higher levels of bone turner markers, which indicated a role for the *DIO2* gene in the regulation of bone formation [8]. Recent research has also shown that single nucleotide polymorphisms (SNPs) of *DIO2* influence thyroid metabolism, resulting in neurological disease [9]. Thyroid hormones (THs) have pivotal roles in the development and growth of the craniofacial skeleton, and their action in target tissues is dependent on the intracellular concentration of T3, which is locally regulated by *DIO2* and deiodinase 3 (*DIO3*) [10]. To date, the regulatory mechanisms of craniofacial malformation caused by TH deficiency or excess have mainly focused on mutations of a single gene [11,12]. As a key gene regulating the synthesis of THs, it was not clear whether the *DIO2* gene affected the development and growth of the craniofacial skeleton.

As an economically important poultry, domestic geese have distinct appearance characteristics, especially in the head. Recent studies showed that the *DIO2* gene was considered the crucial gene associated with geese’s knob phenotype, a feature located at the base of the upper bill in Chinese native geese breeds and absent in European geese breeds, and the growth and development of the knob was mediated by the TH synthesis signaling pathway in geese [13,14]. Notably, the knob has impressive value, as a large knob phenotypic size is generally preferred in Chinese markets according to market research [13]. Therefore, the aim of the present study was to identify and compare the molecular characteristics of the *DIO2* gene between Sichuan White geese (with a knob) and Landes geese (devoid of a knob) and then to analyze the association between polymorphisms of the *DIO2* gene and the craniofacial dimensions in the Tianfu meat geese breed, which was constructed by crossing Sichuan White geese and Landes geese. This study was approved by the 6th (2015) China Waterfowl Development Conference. These results contribute to basic research on the craniofacial characteristics of geese and further breeding for the Tianfu meat geese breed.

## MATERIALS AND METHODS

### Ethics approval

This study was conducted according to the guidelines of the Institutional Animal Care and Use Committee (IACUC) of Sichuan Agricultural University (Chengdu campus, Sichuan, China, Permit No. DKY20170913).

### Animals and sample preparation

Sichuan White geese, Landes geese, and Tianfu meat geese used in the present study were provided by the Waterfowl Breeding Experimental Farm of Sichuan Agricultural University (Ya’an, Sichuan). The population of Tianfu meat geese has been artificially selected for over 10 generations and the craniofacial characteristics are significantly different. The geese were raised in floor pens with free access to water and food. Hypothalamus tissues from Sichuan White geese (3 years old, n = 4) and Landes geese (3 years old, n = 4) were used to clone the coding sequence of the *DIO2* gene. Blood samples (5 mL/goose) from Tianfu meat geese were collected from the wing veins of all geese (3 years old, n = 350, 75 males; 275 females) into ethylenediaminetetraacetic acid-coated tubes for deoxyribonucleic acid (DNA) extraction.

### Ribonucleic acid extraction and molecular cloning

Total ribonucleic acid (RNA) was isolated from the hypothalamus of Sichuan White geese and Landes geese using TRIzol reagent (Takara, Dalian, China). The purity and quality of RNA were detected by spectrophotometric absorbance at 260/280 nm and 260/230 nm, respectively. The integrity of RNA was verified on a 1.5% agarose gel. Complementary DNA (cDNA) was synthesized from 1 μg of total RNA using a cDNA synthesis kit (Takara, China) according to the manufacturer’s instructions. The polymerase chain reaction (PCR) was performed in a total volume of 10 μL using 5 μL of 2× PCR HeroTM Mix(dye) (FOREGENE, Chengdu, China), 1 μL of cDNA, 0.2 μL of primers (10 μM each) and 3.6 μL of ddH_2_O. PCR was performed as follows: 94°C predenaturation for 3 min, 35 cycles at 94°C denaturation for 10 s, annealing temperature for 10 s, and 72°C extension for 20 s, with a final extension at 72°C for 5 min. The PCR products were gel-purified using a gel extraction kit (OMEGA, Norwalk, CT, USA). Target cDNA was ligated into the pMD-19T vector (Takara, China) and was then transformed into *Escherichia coli* DH5α competent cells. Positive clones that contained the expected-size inserts were screened by using colony PCR and were then sequenced by Qinke Gene Biotechnology Co. Ltd. (Chengdu, China). Primers of the *DIO2* gene are shown in Supplementary Table S1.

### Bioinformatical analysis

VecScreen (https://www.ncbi.nlm.nih.gov/tools/vecscreen/) was used to search for vector contamination. The BLASTn program in the National Center for Biotechnology Information (NCBI) was used to analyze the accuracy of cloning sequences (https://blast.ncbi.nlm.nih.gov/Blast.cgi). DNAMAN software was used to analyze the homologies of nucleotide sequences and to identify the polymorphic loci of the *DIO2* gene between Sichuan White geese and Landes geese by sequence alignment. MEGA 7.0 software was used to construct a phylogenetic tree by the neighbor-joining method with 1,000 bootstrap replicates.

### Polymorphic loci validation and genotype in Tianfu meat geese

Tianfu meat geese needed to continue to be used in production; therefore, genomic DNA of each individual was extracted from whole blood using an Animal Tissue DNA Isolation Kit (DE-05012, FOREGENE, China) following the manufacturer’s protocol. Three pairs of primers were designed for screening the polymorphic loci located at the coding sequence of the *DIO2* gene in Tianfu meat geese (Supplementary Table S1). The reaction system was performed in a total volume of 20 μL using 10 μL of 2× PCR HeroTM Mix(dye) (FOREGENE, China), 2 μL of DNA, 0.4 μL of primers (10 μM each) and 7.2 μL of ddH_2_O. The PCR conditions were 94°C predenaturation for 3 min, 35 cycles at 94°C denaturation for 10 s, annealing temperature for 10 s, 72°C extension 20 s, and a final extension at 72°C for 5 min. The PCR products were examined by 1.5% agarose gel electrophoresis and then sequenced by Qinke Gene Biotechnology Co. Ltd. (China).

### The measurement of head dimensions in Tianfu meat geese

**Figure 1:**
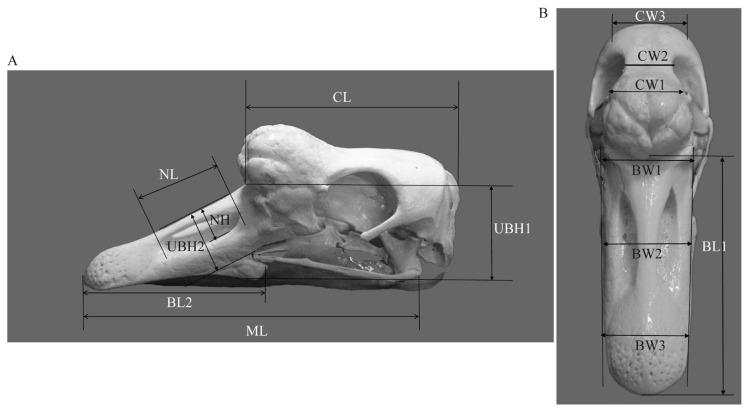
Length, height and width measurement of the goose head. (A) Lateral aspect image. CL, cranial length; ML, maxillary length; BL2, beak length 2; NL, nostril length; UBH1, height of upper beak 1; UBH2, height of upper beak 2; NH, nostril height. (B) Dorso-ventral aspect image. BL1, beak length 1; CW1, cranial width 1; CW2, cranial width 2; CW3, cranial width 3; BW1, beak width 1; BW2, beak width 2; BW3, beak width 3.

The body weight of Tianfu meat geese was recorded, and the head dimensions were measured individually. The measurement standards of head dimensions are shown in Figure 1A and 1B. These indices included cranial length (CL, length from occipital bone to the junction between the frontal bone and the nasal bone), maxillary length (ML, length from the end of the quadratojugal bone to the tip of the beak), beak length 2 (BL2, length from the tip of the quadratojugal bone to the tip of the beak), nostril length (NL, length between the upper and lower nostril), nostril height (NH, height of the center of the nasal bone perpendicular to the nostril), height of upper beak 1 (UBH1, height of the junction between the frontal bone and the nasal bone to the tip of the quadratojugal bone), height of upper beak 2 (UBH2, height of the center of the nasal bone perpendicular to the upper beak), beak length 1 (BL1, length from the junction between the frontal bone and the nasal bone to the tip of the beak), cranial width 1 (CW1, breadth of the junction across the orbits and the frontal bone), cranial width 2 (CW2, smallest breadth between the orbits on the dorsal side), cranial width 3 (CW3, breadth across the protuberentia occipitalis externa), beak width 1 (BW1, breadth of the junction between the frontal bone and the nasal bone), beak width 2 (BW2, breadth of the center of the nasal bone), and beak width 3 (BW3, breadth across the beak bean). Centimeter (cm) was used as the unit for length, height, and width.

### Statistical analysis

In the present study, Tianfu meat geese individuals whose body weights were more than twice the standard deviations from the mean were removed, and several individuals with uncertain knobs were also eliminated. Ultimately, 304 individuals (60 males; 244 females) were used for further analysis. The sequencing results were viewed in BioEdit software (https://bitesizebio.com/10238/bioedit-a-sequence-alignment-editor-and-it-is-free/) to ensure the polymorphic loci of the *DIO2* gene. The information of allele and genotype of *DIO2* gene was sorted in Excel 2019. Allelic and genotypic frequencies were determined by direct counting. Population indices, including heterozygosity (*He*), homozygosity (*Ho*), and effective number of alleles (*Ne*) were calculated by the following formulas:


$$Ho=\sum_{i=1}^{n}p_{i}^{2};\;He=1-Ho;\;Ne=1/Ho$$

*P_i_* represents the i-th allelic frequency at a locus, and *n* represents the number of alleles at a locus.

The polymorphism information content (PIC) was directly calculated by PIC_CALC 0.6 software. The polymorphic loci of the *DIO2* gene were subjected to linkage disequilibrium (LD) analysis using the SHEsis platform and to haplotype/diplotype analysis by Haploview 4.1. The haplotypes and dipoltypes with frequencies below 5% were not subjected to subsequent analysis.

The general linear model (GLM) procedure of SPSS. 24.0 was used to test the association between the knob phenotype and head dimensions. The model was as follows:


$$Y_{ij}=\mu +{\text{G}}_{i}+{\text{S}}_{j}+{\text{e}}_{ij}$$

where *Y_ij_* is the measurement of a trait, μ is the overall population mean, G*_i_* is the fixed effect of phenotype (i = 2), S*_j_* is the fixed effect of sex (j = 2) and e*_ij_* is the random error. The values are presented as the mean±standard deviation. Multiple comparisons were carried out using analysis of variance followed by Duncan’s test using SPSS. 24.0. An independent nonpaired t-test analysis (two-tailed) was used for comparisons between the two groups. Statistical significance was considered at p<0.05.

## RESULTS

### *DIO2* gene cloning and sequence comparison analysis

**Figure 2:**
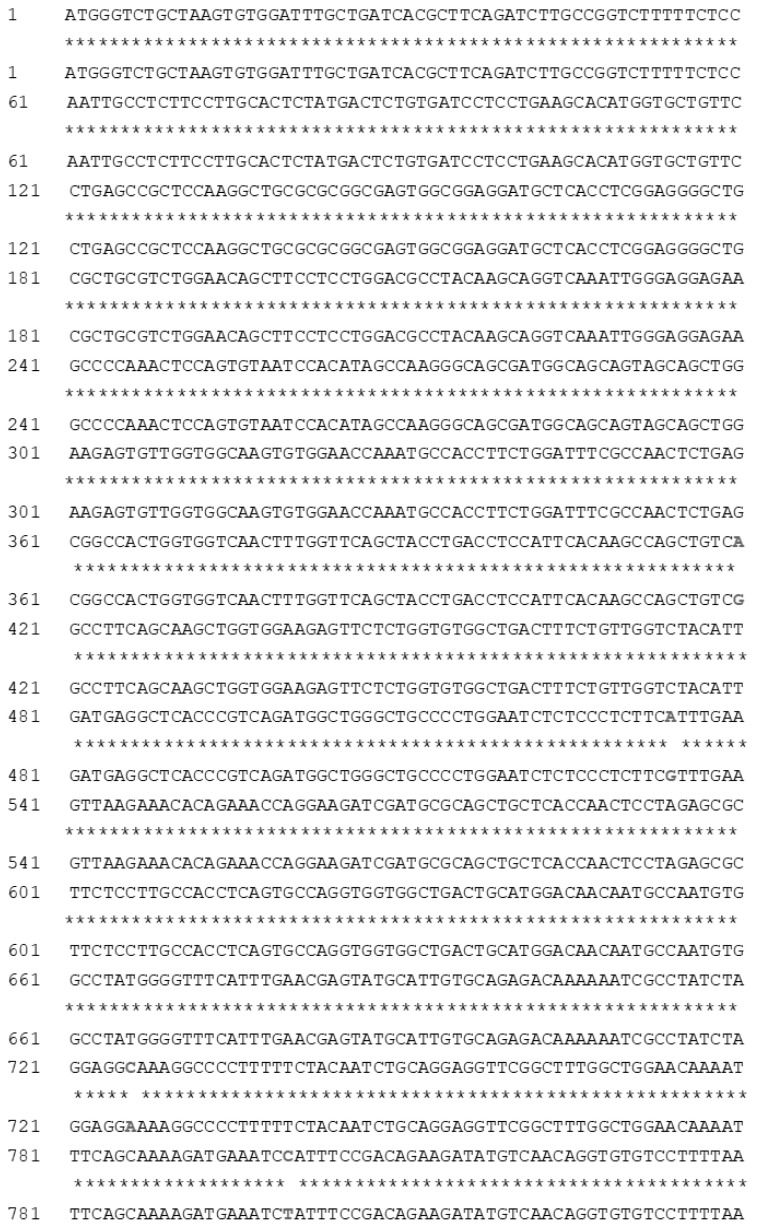
Comparison analysis of the deiodinase 2 gene coding sequences between Sichuan White geese and Landes geese. The top row represented Landes geese, the bottom row represented Sichuan White geese. * Indicated the consistent nucleotides, locations at 419, 533, 725, 799 indicated the inconsistent nucleotides. The number on the left represented the position of nucleotides.

In the present study, the coding sequences of the *DIO2* gene of both Sichuan White geese and Landes geese were cloned. The sequence of the *DIO2* gene consisted of 840 nucleotides (Figure 2). Homology analysis of nucleotide sequences showed that there was high sequence homology (95.42%) between geese and other avian species, such as chicken and quail, intermediate homology (88.06%) between geese and fish, such as zebrafish, and relatively less homology (86.95%) between geese and mammals, such as humans and mice. In addition, a phylogenetic tree was constructed based on the *DIO2* nucleotide acid sequences for geese and other species (Supplementary Figure S1), which indicated that the *DIO2* genes in Sichuan White geese and Landes geese were similar to the *DIO2* genes of other avians. Furthermore, the sequences of the *DIO2* genes of Sichuan White geese and Landes geese were aligned, and four polymorphic loci, g.419 A>G, g.533 A>G, g.725 C>A, and g.799 C>T, were identified. Among these loci, the g.799 C>T locus changed the amino acid from lysine to proline.

### Genetic parameter analysis of *DIO2* gene polymorphisms in Tianfu meat geese

**Figure 3:**
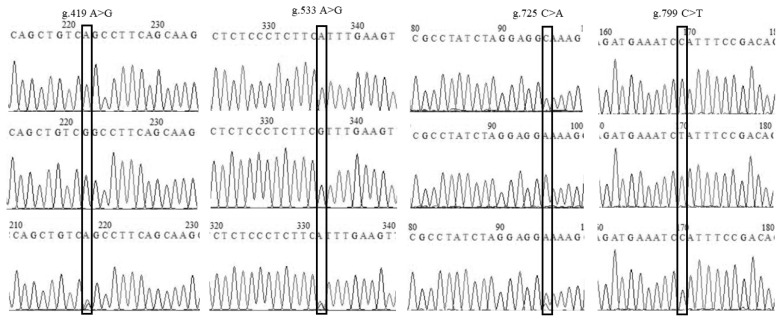
Confirmation of the four polymorphic loci of the deiodinase 2 gene in the population of Tianfu meat geese. The black boxes represented the polymorphic loci. The first and second rows were homozygotes, and the third row was heterozygote.

To identify the genetic parameters of *DIO2* gene polymorphisms, a crossbred population of Tianfu meat geese was used. Genotype analysis showed that the g.419 A>G, g.533 A>G, g.725 C>A, and g.799 C>T loci had three genotypes, AA/GG/GA, AA/GG/GA, CC/AA/AC, and CC/TT/TC, in Tianfu meat geese (Figure 3). The genotypic and allelic frequencies as well as estimated population indices (heterozygosity, effective allele numbers, PIC) were calculated and were shown in Supplementary Table S2. The results showed that the dominant alleles at the g.419A>G, g.533A>G, g.725C>A, and g.799C>T loci were G, G, A, and T, respectively. The frequencies of heterozygous genotypes of four polymorphic loci in this population were higher than those of homozygous genotypes. The estimation of population indices showed that the values of *Ho* and *He* among g.419 A>G, g.533 A>G, g.799 C>T loci were consistent, in which *Ho* was observed as 0.50, *He* was observed as 0.50, and Ne was observed as 1.98, while at the g.725 C>A locus, *Ho* was 0.51, *He* was 0.49, and *Ne* was 1.97. According to the classification of PIC, the four polymorphic loci of the *DIO2* gene in this population possessed intermediate polymorphisms (0<PIC<0.5). The chi-square test showed that the genotypic frequencies of the four polymorphic loci were in accordance with Hardy-Weinberg equilibrium (p> 0.05).

### Association analysis between knob phenotype and the head dimensions of geese

To investigate whether the knob and non-knob phenotypes influenced the head dimensions of geese, a comparison analysis of head dimensions was carried out between knob and non-knob goose populations. The results showed that there was no interaction between the fixed effect of sex and the knob and non-knob phenotypes. Compared to that in the population of non-knob geese, the head dimensions in the population of knob geese were significantly higher except for NH (Table 1, p<0.05).

### Association analysis between polymorphisms of the *DIO2* gene and the head dimensions of geese

As stated in the previous results, the *DIO2* gene was crucial to regulate the knob and non-knob phenotypes, and the knob phenotype was related to the head dimensions of geese. Thus, the population of Tianfu meat geese was divided into populations of knob and non-knob geese, and the association analysis of polymorphisms of the *DIO2* gene with the head dimensions of geese was carried out in populations of knob and non-knob geese. In the population of knob geese, as shown in Table 2, the different genotypes of polymorphisms of the *DIO2* gene were not significantly different from the head dimensions of geese (p>0.05). Further analysis found that individuals with the AA genotype at the g.419A>G and g.533A>G loci or the CC genotype at the g.725C>A and g.799C>T loci had higher BL1, BW1, CW1, ML, UBH2, and NH than those with the GG genotype at the g.419A>G and g.533A>G loci, AA genotype at the g.725C>A locus and TT genotype at the g.799C>T locus (p>0.05). However, in the population of non-knob geese, as shown in Table 3, individuals with the AA genotype at the g.419A>G and g.533A>G loci or the CC genotype at the g.725C>A and g.799C>T loci were significantly higher in BW1, BW2, and NL than in other genotypes (p<0.05). Individuals with the CC genotype at the g.725C>A and g.799C>T loci were significantly wider in CW1 than in other genotypes (p<0.05). At the g.419A>G and g.533A>G loci, individuals with the AA genotype were significantly longer in ML than those with other genotypes, while individuals with the GA genotype were significantly higher in NH than those with other genotypes (p<0.05).

### Association analysis between haplotypes/diplotypes and the head dimensions of geese

The LD test showed that the four polymorphic loci of the *DIO2* gene were strongly linked in the population of non-knob geese (Supplementary Figure S2). The haplotype analysis showed that two different haplotypes were identified: GGAT with 53.3% frequency and AACC with 43.7% frequency (Supplementary Table S3). Based on the haplotype analysis results, three major available diplotypes, GGAT-GGAT, GGAT-AACC, and AACC-AACC, were identified (Supplementary Table S3). Further association analysis showed that individuals with the AACC haplotype were significantly higher in BW1, CW1 and NL than in GGAT (Table 4, p<0.05), while individuals with the GGAT haplotype were significantly higher in NH than in AACC (Table 4, p<0.05). Diplotype analysis showed that individuals with AACC-AACC diplotypes were significantly higher in BW1, BW2, CW1, and NL than in GGAT-AACC and GGAT-GGAT diplotypes (Table 4, p< 0.05), while individuals with GGAT-AACC were significantly higher in NH than in AACC-AACC and GGAT-GGAT diplotypes (Table 4, p<0.05).

## DISCUSSION

The *DIO2* gene is responsible for converting prohormone T4 to the active hormone T3 and plays important roles in the synthesis of TH and the process of development. To date, the full-length coding sequence of the *DIO2* gene has been cloned in humans [15], chickens [16], mice [17], and bovines [18] to study its expression level and regulation of TH. In the present study, the coding sequences of the *DIO2* gene in Sichuan White geese (knob geese) and Landes geese (non-knob geese) were cloned and had the closest homology with avians, which indicated a similar function between geese and other avians.

Robinson et al [19] showed that the appearance phenotypes of animals were associated with their growth, reproduction and other biological functions. For instance, an advantage in the fertility of horned males was found compared to polled males [20]. The various combs and wattles in chickens were related to egg weight, thermoregulation and sperm motility [21–23]. In the present study, individuals with the presence of a knob had greater head dimensions than those with the absence of a knob, which indicated that the knob phenotype might influence the skeletal development of the head in geese. Our previous results showed that the *DIO2* gene played a pivotal role in determining the knob phenotype in geese and that a nonsynonymous mutation was identified in knob geese [14]. In the present study, four polymorphic loci of the *DIO2* gene were found by comparison analysis of coding sequences between Sichuan White geese and Landes geese, which were further genotyped in Tianfu meat geese and three genotypes in each locus were identified. The values of *Ho*, *He*, and *Ne* reflected the degree of genetic variation in the population, and PIC represented the genetic information content [24]. Therefore, based on the analysis of the population indices and PIC value (Supplementary Table S2), our results showed that polymorphic loci of the *DIO2* gene were at an intermediate level of genetic diversity. Furthermore, the dominant alleles at the g.419A>G, g.533A>G, g.725C>A, and g.799C>T loci were G, G, A, and T, respectively, and the frequencies of heterozygous genotypes of the four loci were higher than those of homozygous genotypes (Supplementary Table S2). Notably, the population of Tianfu meat geese was mainly used for breeding in growth and reproductive performance, while the knob phenotype was not the breeding target of this population. Therefore, the higher frequencies of heterozygous genotypes might be related to the artificial selection of the population, which is an important factor that can affect gene equilibrium in domestic animal populations [25]. Mentuccia et al [26] first identified a polymorphism of the *DIO2* gene (Thr92Ala), and studies of Thr92Ala in the *DIO2* gene were carried out in humans and mainly focused on its associations with diseases, such as Alzheimer’s disease [27] and Kashin-Beck disease [28]. Another study also showed that the rs225017 polymorphism in the 3′UTR of the human *DIO2* gene was associated with greater insulin resistance (IR) and interacted with the Thr92Ala polymorphism in the modulation of IR [29]. In the present study, the polymorphic loci of the *DIO2* gene were significantly associated with BW1, BW2, NL, CW1, and ML in the population of non-knob geese. Moreover, the polymorphic loci of the *DIO2* gene had a strong linkage association, and two haplotypes and three diplotypes were identified in the population of non-knob geese. Fallin et al [30] showed that studying the inheritance of haplotypes was more important than studying individual SNPs. In the present study, individuals with AACC haplotype/AACC-AACC diplotype were significantly higher in BW1, CW1 and NL than in GGAT haplotype/GGAT-AACC and GGAT-GGAT diplotypes. These results suggested that these polymorphic loci of the *DIO2* gene might be used as molecular markers to select larger heads in the population of non-knob geese.

In conclusion, the head dimensions of geese were signifi cantly different between knob and non-knob geese, and polymorphic loci of the *DIO2* gene could be considered as molecular markers to select larger heads of geese in the population of non-knob geese.

## Figures and Tables

**Table 1:** Association analysis between the knob and the head dimensions in goose

Knob types	BL1	BL2	BW1	BW2	BW3	CW1	CW2	CW3	ML	CL	UBH1	UBH2	NL	NH
Knob geese (138)	6.62±0.51^*^	7.19±0.57	3.18±0.21^*^	2.74±0.17^*^	2.51±0.15^*^	2.89±0.26^*^	2.29±0.22^*^	3.08±0.24^*^	13.80±0.91^*^	8.16±0.45^*^	3.13±0.27^*^	1.88±0.25^*^	2.91±0.24^*^	0.81±0.15
Non-knob geese (166)	6.29±0.39	6.89±0.46	3.01±0.18	2.61±0.14	2.41±0.10	2.73±0.18	2.16±0.19	2.94±0.25	13.25±0.66	7.91±0.36	3.01±0.24	1.78±0.19	2.83±0.22	0.80±0.14

The number behind the knob types represented the number of individuals. Centimeter (cm) was used as the unit for length, height and width.

BL1, beak length 1; BL2, beak length 2; BW1, beak width 1; BW2, beak width 2; BW3, beak width 3; CW1, cranial width 1; CW2, cranial width 2; CW3, cranial width 3; ML, maxillary length; CL, cranial length; UBH1, height of upper beak 1; UBH2, height of upper beak 2; NL, nostril length; NH, nostril height.

* Represented the statistical significance within the same column differ significantly at p<0.05.

**Table 2:** Association analysis between polymorphic loci of the deiodinase 2 gene and the head dimensions in knob geese

Loci	Genotypes	BL1	BL2	BW1	BW2	BW3	CW1	CW2	CW3	ML	CL	UBH1	UBH2	NL	NH
g.419 A>G	GG (40)	6.65±0.46	7.23±0.50	3.17±0.20	2.74±0.18	2.51±0.16	2.85±0.21	2.25±0.20	3.07±0.22	13.77±0.85	8.19±0.39	3.17±0.27	1.88±0.20	2.92±0.21	0.80±0.16
	AA (27)	6.67±0.52	7.19±0.60	3.20±0.20	2.71±0.15	2.51±0.14	2.93±0.27	2.24±0.25	3.06±0.27	13.89±0.88	8.11±0.48	3.15±0.29	1.90±0.27	2.92±0.22	0.82±0.19
	GA (71)	6.59±0.54	7.17±0.61	3.18±0.21	2.75±0.18	2.52±0.15	2.90±0.29	2.33±0.22	3.09±0.25	13.78±0.96	8.16±0.48	3.10±0.27	1.87±0.27	2.90±0.26	0.82±0.15
g.533 A>G	GG (40)	6.65±0.46	7.23±0.50	3.17±0.20	2.74±0.18	2.51±0.16	2.85±0.21	2.25±0.20	3.07±0.22	13.77±0.85	8.19±0.39	3.17±0.27	1.88±0.20	2.92±0.21	0.80±0.16
	AA (27)	6.67±0.52	7.19±0.60	3.20±0.20	2.71±0.15	2.51±0.14	2.93±0.27	2.24±0.25	3.06±0.27	13.89±0.88	8.11±0.48	3.15±0.29	1.90±0.27	2.92±0.22	0.82±0.19
	GA (71)	6.59±0.54	7.17±0.61	3.18±0.21	2.75±0.18	2.52±0.15	2.90±0.29	2.33±0.22	3.09±0.25	13.78±0.96	8.16±0.48	3.10±0.27	1.87±0.27	2.90±0.26	0.82±0.15
g. 725 C>A	AA (43)	6.59±0.52	7.20±0.51	3.15±0.21	2.73±0.18	2.49±0.16	2.85±0.20	2.26±0.19	3.07±0.23	13.70±0.89	8.15±0.42	3.12±0.31	1.85±0.24	2.88±0.24	0.80±0.16
	CC (25)	6.67±0.50	7.25±0.55	3.21±0.21	2.72±0.15	2.51±0.14	2.95±0.27	2.25±0.26	3.09±0.27	13.89±0.90	8.13±0.47	3.16±0.30	1.91±0.28	2.93±0.23	0.82±0.19
	AC (70)	6.64±0.51	7.19±0.60	3.19±0.21	2.76±0.18	2.53±0.14	2.90±0.29	2.32±0.22	3.08±0.25	13.83±0.93	8.17±0.47	3.12±0.25	1.88±0.25	2.92±0.25	0.82±0.14
g.799 C>T	TT (41)	6.62±0.51	7.23±0.50	3.16±0.21	2.75±0.18	2.51±0.17	2.85±0.20	2.26±0.19	3.08±0.22	13.73±0.88	8.20±0.41	3.16±0.28	1.89±0.20	2.92±0.21	0.80±0.16
	CC (26)	6.64±0.51	7.19±0.62	3.20±0.21	2.72±0.15	2.51±0.14	2.93±0.27	2.24±0.25	3.08±0.27	13.87±0.89	8.14±0.47	3.15±0.30	1.90±0.27	2.92±0.23	0.82±0.19
	TC (71)	6.62±0.52	7.17±0.61	3.19±0.21	2.75±0.18	2.52±0.14	2.90±0.29	2.32±0.22	3.08±0.25	13.81±0.94	8.14±0.48	3.10±0.26	1.85±0.27	2.90±0.26	0.82±0.14

The number behind the genotype represented the number of individuals. Centimeter (cm) was used as the unit for length, height and width.

BL1, beak length 1; BL2, beak length 2; BW1, beak width 1; BW2, beak width 2; BW3, beak width 3; CW1, cranial width 1; CW2, cranial width 2; CW3, cranial width 3; ML, maxillary length; CL, cranial length; UBH1, height of upper beak 1; UBH2, height of upper beak 2; NL, nostril length; NH, nostril height.

**Table 3:** Association analysis between polymorphic loci of the deiodinase 2 gene and the head dimensions in non-knob geese

Loci	Genotypes	BL1	BL2	BW1	BW2	BW3	CW1	CW2	CW3	ML	CL	UBH1	UBH2	NL	NH
g.419 G>A	GG (56)	6.32±0.39	6.92±0.47	3.00±0.18	2.60±0.15	2.40±0.10	2.70±0.21	2.15±0.19	2.96±0.23	13.31±0.67	7.91±0.39	3.02±0.24	1.77±0.18	2.82±0.21	0.80±0.13
	AA (40)	6.33±0.38	6.92±0.39	3.09±0.19^*^	2.66±0.15^*^	2.43±0.12	2.79±0.16	2.19±0.19	2.91±0.31	13.39±0.69^*^	7.99±0.32	3.03±0.26	1.75±0.17	2.92±0.19^*^	0.76±0.10
	GA (70)	6.25±0.39	6.86±0.49	2.98±0.16	2.59±0.13	2.40±0.10	2.72±0.17	2.16±0.20	2.95±0.23	13.13±0.61	7.86±0.35	2.98±0.24	1.80±0.21	2.79±0.23	0.82±0.16^*^
g.533 G>A	GG (56)	6.32±0.39	6.92±0.47	3.00±0.18	2.60±0.15	2.40±0.10	2.70±0.21	2.15±0.19	2.96±0.23	13.31±0.67	7.91±0.39	3.02±0.24	1.77±0.18	2.82±0.21	0.80±0.13
	AA (40)	6.33±0.38	6.92±0.39	3.09±0.19^*^	2.66±0.15^*^	2.43±0.12	2.79±0.16	2.19±0.19	2.91±0.31	13.39±0.69^*^	7.99±0.32	3.03±0.26	1.75±0.17	2.92±0.19^*^	0.76±0.10
	GA (70)	6.25±0.39	6.86±0.49	2.98±0.16	2.59±0.13	2.40±0.10	2.72±0.17	2.16±0.20	2.95±0.23	13.13±0.61	7.86±0.35	2.98±0.24	1.80±0.21	2.79±0.23	0.82±0.16^*^
g. 725 A>C	AA (56)	6.32±0.39	6.91±0.47	2.99±0.18	2.61±0.14	2.41±0.11	2.70±0.21	2.16±0.19	2.97±0.23	13.25±0.63	7.94±0.36	3.02±0.25	1.78±0.17	2.81±0.21	0.81±0.13
	CC (39)	6.32±0.38	6.91±0.39	3.09±0.19^*^	2.65±0.15^*^	2.43±0.11	2.78±0.16^*^	2.19±0.18	2.91±0.31	13.36±0.68	7.99±0.32	3.04±0.26	1.75±0.17	2.92±0.19^*^	0.76±0.10
	AC (71)	6.25±0.39	6.87±0.48	2.99±0.15	2.59±0.13	2.40±0.10	2.72±0.17	2.15±0.20	2.94±0.23	13.19±0.66	7.85±0.37	2.98±0.23	1.79±0.21	2.80±0.23	0.81±0.16
g.799 T>C	TT (55)	6.33±0.38	6.94±0.46	3.01±0.18	2.62±0.14	2.41±0.11	2.71±0.21	2.16±0.19	2.96±0.23	13.32±0.66	7.92±0.39	3.02±0.24	1.78±0.18	2.82±0.21	0.81±0.13
	CC (39)	6.32±0.38	6.91±0.39	3.09±0.19^*^	2.65±0.15^*^	2.43±0.11	2.78±0.16^*^	2.19±0.18	2.91±0.31	13.36±0.68	7.99±0.32	3.04±0.26	1.75±0.17	2.92±0.19^*^	0.76±0.10
	TC (72)	6.24±0.40	6.84±0.49	2.98±0.16	2.58±0.13	2.39±0.10	2.72±0.17	2.15±0.20	2.95±0.23	13.13±0.63	7.86±0.34	2.98±0.24	1.79±0.21	2.79±0.23	0.81±0.14

The number behind the genotype represented the number of individuals. Centimeter (cm) was used as the unit for length, height and width.

BL1, beak length 1; BL2, beak length 2; BW1, beak width 1; BW2, beak width 2; BW3, beak width 3; CW1, cranial width 1; CW2, cranial width 2; CW3, cranial width 3; ML, maxillary length; CL, cranial length; UBH1, height of upper beak 1; UBH2, height of upper beak 2; NL, nostril length; NH, nostril height.

* Represented the statistical significance, p<0.05.

**Table 4:** Association analysis between haplotype/diplotype of the deiodinase 2 gene and the head dimensions in the population of non-knob geese

Hap/Dip	BL1	BL2	BW1	BW2	BW3	CW1	CW2	CW3	ML	CL	UBH1	UBH2	NL	NH
GGAT	6.35±0.39	6.94±0.47	3.00±0.18	2.61±0.14	2.40±0.10	2.70±0.21	2.16±0.19	2.97±0.23	13.27±0.64	7.93±0.37	3.03±0.24	1.78±0.18	2.82±0.22	0.81±0.13^*^
AACC	6.32±0.38	6.91±0.39	3.09±0.19^*^	2.65±0.15	2.43±0.11	2.78±0.16^*^	2.19±0.18	2.91±0.31	13.36±0.68	7.99±0.32	3.04±0.26	1.75±0.17	2.92±0.19^*^	0.76±0.10
AACC-AACC	6.32±0.38	6.91±0.39	3.09±0.19^*^	2.65±0.15^*^	2.43±0.11	2.78±0.16^*^	2.19±0.18	2.91±0.31	13.36±0.68	7.99±0.32	3.04±0.26	1.75±0.17	2.92±0.19^*^	0.76±0.10
GGAT-AACC	6.26±0.40	6.86±0.49	2.99±0.16	2.59±0.12	2.39±0.09	2.72±0.17	2.15±0.20	2.95±0.23	13.12±0.62	7.85±0.36	2.99±0.24	1.80±0.22	2.79±0.24	0.82±0.17^*^
GGAT-GGAT	6.35±0.39	6.94±0.47	3.00±0.18	2.61±0.14	2.40±0.10	2.70±0.21	2.16±0.19	2.97±0.23	13.27±0.64	7.93±0.37	3.03±0.24	1.78±0.18	2.82±0.22	0.81±0.13

Centimeter (cm) was used as the unit for length, height and width.

BL1, beak length 1; BL2, beak length 2; BW1, beak width 1; BW2, beak width 2; BW3, beak width 3; CW1, cranial width 1; CW2, cranial width 2; CW3, cranial width 3; ML, maxillary length; CL, cranial length; UBH1, height of upper beak 1; UBH2, height of upper beak 2; NL, nostril length; NH, nostril height.

* Represented the statistical significance, p<0.05.
