# Supplementary material for: Identification of polymorphic loci in the deiodinase 2 gene and their associations with head dimensions in geese
Source: Anim Biosci. 2021 Oct 29;35(5):639–47. doi: 10.5713/ab.21.0382 (PMC9065781; doi:10.5713/ab.21.0382)
Supplement: Supplementary file 1 [file ab-21-0382-suppl.pdf]

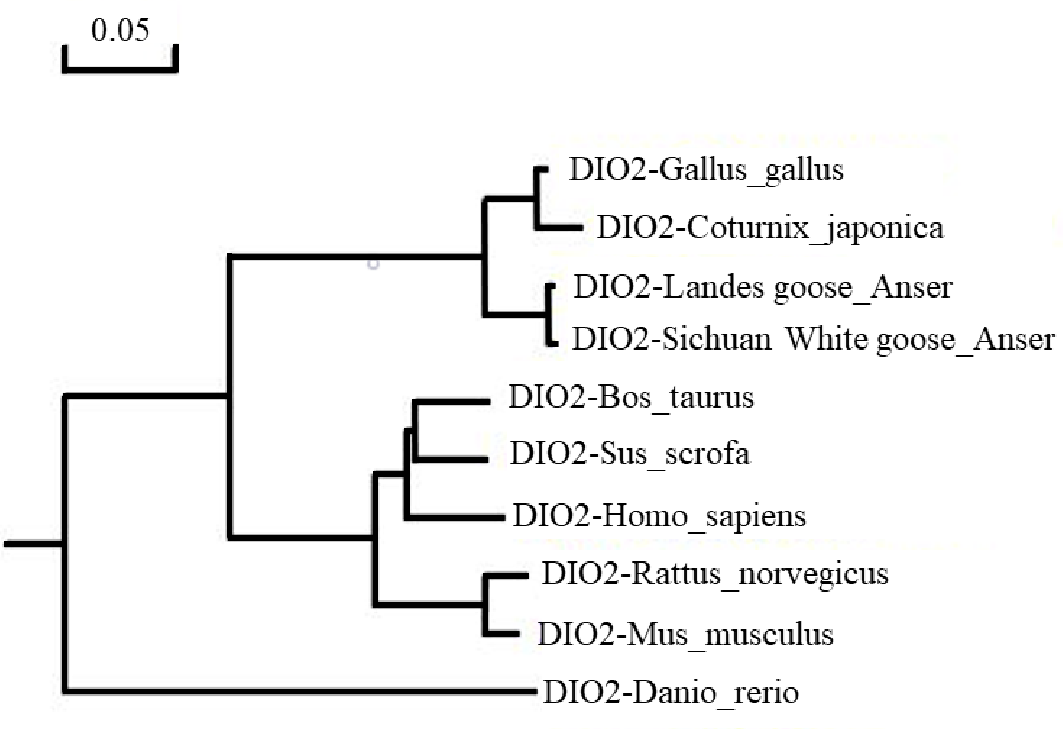

Supplementary Figure 1

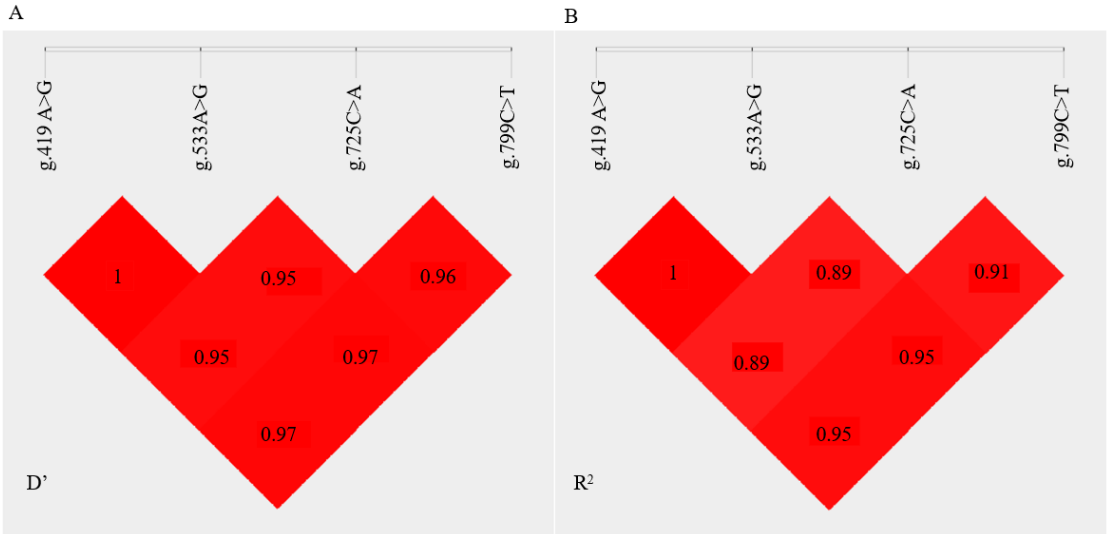
A
g.419 A>G
g.533A>G
g.725C>A
g.799C>T
1
0.95
0.96
0.95
0.97
0.97
D'
B
g.419 A>G
g.533A>G
g.725C>A
g.799C>T
1
0.89
0.91
0.89
0.95
0.95
R2

Supplementary Table 1. Information of primer used for PCR

Primer sequence (5’ - 3’)	Length (bp)	Annealed temperature (℃)	Genbank (ID)
For coding sequence			
F: CTAAGTGAGGGCATTGGGC<br>R: AGCCATCTGACGGGTGAGCCT	571	61	XM_013194617.1
F: CTGACCTCCATTCACAAGCCA<br>R: CTCACCACTCTTCCTGCTCAAC	706	59	
For mutation loci			
F: CTAAGTGAGGGCATTGGGC<br>R: CACGGAGTGCGAGCAGTAG	523	58	
F: CTGGACAAGCAAGTCGCAC<br>R: CAGCCAAAGCCGAACCTC	602	58	

F: GCTTCTCCTTGCCACCTC<br>R: CACTCTTCCTGCTCAACTGC	495	55

F, forward sequence; R, reverse sequence.

Supplementary Table 2. Genotypic and allelic frequencies and population indexes for three single nucleotide polymorphisms (SNPs) of the goose *DIO2* gene

Position	Observed	Genotype	Allelic	χ^2		Genetic polymorphism		
	Genotypes (N)	frequencies	frequencies	(*P*-value)	PIC	He	Ho	Ne
g.419 A>G	GG (96)	0.32	0.548 (G)	1.24	0.373	0.495	0.505	1.98
	AA (67)	0.22	0.452 (A)	(p = 0.27)				
	GA (141)	0.46						
g.533 A>G	GG (96)	0.32	0.548 (G)	1.24	0.373	0.495	0.505	1.98
	AA (67)	0.22	0.452 (A)	(p = 0.27)				
	GA (141)	0.46						
g.725 C>A	AA (99)	0.33	0.558 (A)	1.22	0.372	0.493	0.507	1.97
	CC (64)	0.21	0.442 (C)	(p = 0.27)				

	AC (140)	0.46						
	TT (96)	0.32	0.551 (T)	0.74				
g.799 C>T	CC (65)	0.21			0.372	0.495	0.505	1.98
			0.449 (C)	(*p* = 0.39)				
	TC (143)	0.47						

Note: Numbers in parentheses indicated the number of individuals. PIC: polymorphism information content. He: heterozygosity. Ho: homozygosity. Ne: effective number of alleles.

Supplementary Table 3. The frequencies of each haplotypes/diplotypes for four loci of goose *DIO2* gene

Haplotype/Diplotype	Frequency (%)
GGAT	53.3
AACC	43.7
GGAT-GGAT	30.72
GGAT-AACC	39.76
AACC-AACC	23.49
